# Extremely early initiation of vasopressors might not decrease short-term mortality for adults with septic shock: a systematic review and meta-analysis

**DOI:** 10.1186/s13613-025-01428-0

**Published:** 2025-01-27

**Authors:** Cheng-Hsin Ma, Jack Healy, Ebrima Kinteh, Cheng-Chin Ma, Ching-Fang Tiffany Tzeng, Eric H. Chou, Chin-Chieh Wu, Shih-Chieh Shao, Kuan-Fu Chen

**Affiliations:** 1 Department of Medical Education, Linkou Chang Gung Memorial Hospital, 5 Fu-Shin Street, Gueishan district, Taoyuan, 333 Taiwan; 2 Burnett School of Medicine, Texas Christian University, Fort Worth, TX USA; 3 Department of Emergency Medicine, Baylor Scott & White All Saints Medical Center, Fort Worth, TX USA; 4 Department of Artificial Intelligence, College of Intelligent Computing, Chang Gung University, Taoyuan, Taiwan; 5 School of Pharmacy, Institute of Clinical Pharmacy and Pharmaceutical Sciences, College of Medicine, National Cheng Kung University, Tainan, Taiwan; 6 Department of Pharmacy, Keelung Chang Gung Memorial Hospital, Keelung, Taiwan; 7 Department of Emergency Medicine, Keelung Chang Gung Memorial Hospital, Keelung, Taiwan; 8 Department of Emergency Medicine , Linkou Chang Gung Memorial Hospital, 5 Fu-Shin Street, Gueishan district, Taoyuan, 333 Taiwan; 9 Weil Institute, University of Michigan, Ann Arbor, United States; 10 Department of Emergency Medicine, Ohio State University, Columbus, United States

**Keywords:** Sepsis, Septic shock, Resuscitation, Vasopressor therapy, Critical care, Systematic review, Meta-analysis

## Abstract

**Background:**

The optimal timing for initiating vasopressor therapy in patients with septic shock remains unclear. This study aimed to assess the impact of early versus late vasopressor initiation on clinical outcomes.

**Methods:**

A systematic review and meta-analysis were conducted by searching PubMed, Embase, and Cochrane databases. Studies comparing early and late vasopressor administration in septic shock patients were included. The primary outcome was short-term mortality, and subgroup analyses were performed based on different initiation timings.

**Results:**

Eleven studies with 6,661 patients were included. Different studies define the ‘early administration’ timeframe variously, ranging from one to seven hours. No significant difference in short-term mortality was observed between early and late administration in the combined analysis of 3,757 patients from two RCTs and three quasi-experimental studies (OR: 0.66, 95% CI: [0.36, 1.19], I²: 82%). However, lower mortality was found in subgroups with early but not extremely early initiation (one to three hours, OR: 0.70, 95% CI: [0.60, 0.82], I²: 0%), and those using septic shock diagnosis as time zero (OR: 0.64, 95% CI: [0.48, 0.85], I²: 39%).

**Conclusion:**

Our findings found that earlier initiation of vasopressor therapy, particularly within one to three hours after the diagnosis of septic shock, may be associated with reduced short-term mortality in certain subgroups. However, due to the heterogeneity in study definitions and potential confounding factors, these results should be interpreted cautiously. Further standardized investigations are warranted to precisely determine the optimal timing for vasopressor initiation to maximize survival outcomes in patients with septic shock.

**Supplementary Information:**

The online version contains supplementary material available at 10.1186/s13613-025-01428-0.

## Background

Sepsis is a life-threatening condition characterized by a dysregulated response to infection, leading to organ dysfunction. It is a significant global health concern, with an average 30-day mortality of 20–30% [1, 2]. Septic shock, a severe progression of sepsis, is characterized by profound cellular, metabolic, or circulatory abnormalities leading to severe end-organ perfusion, with an increased 30-day mortality rate ranging from 30 to 40% [1, 2]. Prompt and effective management of septic shock is crucial to improve patient outcomes and minimize the risk of morbidity and mortality.

According to the “Surviving Sepsis Campaign: International Guidelines for Management of Sepsis and Septic Shock 2021”, immediate treatment and resuscitation are strongly recommended for patients with septic shock [3]. The guidelines advise initiating vasopressor therapy if fluid resuscitation alone does not achieve adequate perfusion or if hypoperfusion persists. Norepinephrine is specified as the first-line vasopressor; however, the guidelines do not offer specific recommendations on the timing of its initiation. While the importance of immediate treatment and resuscitation in septic shock is well-established, the optimal timing for initiating vasopressors remains uncertain.

The timing of vasopressor initiation is critical, as it can impact patient outcomes. Several studies have explored the association between the timing of vasopressor initiation and short-term mortality in septic shock, but the findings have been conflicting [4–7]. Some studies suggested that delayed administration of norepinephrine, a commonly used vasopressor, is associated with increased short-term mortality [4, 5]. Conversely, other studies indicate that initiating vasopressors within one hour after fluid resuscitation may be associated with higher 28-day mortality [6]. Furthermore, some investigations found no significant association between the time to vasopressor initiation and short-term mortality [7].

These discrepancies highlight the need for a comprehensive analysis to determine the optimal timing for initiating vasopressors in septic shock. One previous systematic review and meta-analysis published in 2020 revealed that early initiation of norepinephrine in patients with septic shock was associated with decreased short-term mortality, shorter time to achieve target MAP, and less volume of intravenous fluids within 6 h [8]. However, limitations such as small sample sizes, heterogeneity, and missing outcomes prevent this study from being widely adopted in clinical practice.

The primary objective of this study was to conduct a systematic review and meta-analysis to determine the optimal timing for vasopressor initiation in septic shock patients.

## Methods

### Protocol

We conducted this systematic review and meta-analysis in accordance with the Preferred Reporting Items for Systematic Reviews and Meta-Analyses (PRISMA 2020) statement [9]. The review protocol was registered with PROSPERO, the International Prospective Register of Systematic Reviews (registration ID: CRD42023388153, registration date: 13 January 2023) [10] to ensure transparency and replicability. As this study was a review of published literature, it did not involve direct research on subjects and did not require approval from the institutional review board.

### Search strategy

We searched the PubMed, Embase, and Cochrane databases for studies that evaluate the optimal timing of starting vasopressors in patients with septic shock, published between January 2010 and November 2023. The following search terms were used: septic shock, vasoconstrictor agents, hypertensive agent, vasopressor, norepinephrine, noradrenalin, time, timing, and initiation (Additional file 1: Table S1, S2, S3). We also conducted a manual review of the reference lists of identified articles. Each title and abstract were screened by at least two investigators (C. H. Ma, J. Healy, E. Kinteh, C. C. Ma) before being included in full-text reviews. Conflicts regarding the inclusion or exclusion of studies for full-text review were resolved through discussion, with a third investigator (K. F. Chen) making the final decision if necessary. The same screening steps were applied to the full-text review, and the final selection of included articles was determined through consensus meetings.

### Study selection

Eligible articles were selected based on the following criteria: (1) adult patients (> 18 years) diagnosed with septic shock; (2) studies comparing different timings of vasopressor initiation in intervention and control groups; (3) outcomes including short-term mortality; and (4) study design comprising RCTs and observational studies. We excluded conference meeting abstracts, review articles, case reports, and non-human studies.

### Data extraction

At least two reviewers (C. H. Ma, J. Healy, E. Kinteh, C. C. Ma) extracted data for each study. We extracted the lead author, year of study conduct, study design, population location, predominant vasopressors, and the definition of early and late groups. The following characteristics were also collected: general demographics, including age and gender, major comorbidities, mortality, and ICU length of stay.

### Types of outcome measures

The primary outcome was short-term mortality, which included in-hospital mortality, 28-day mortality, and 30-day mortality. The secondary outcome was the length of stay in the intensive care unit (ICU).

### Risk of bias assessment

We used the Risk of Bias 2 (RoB 2.0) tool and the Risk of Bias in Non-randomized Studies of Interventions (ROBINS-I) assessment tool to evaluate the risk of bias in the included randomized controlled trials (RCTs) and non-randomized studies, respectively. Similarly, two reviewers (C. H. Ma, J. Healy, E. Kinteh, C. C. Ma) assessed the risk of bias. Any conflicts were resolved by a third investigator (K. F. Chen) who made the final decision if necessary.

### Quantitative synthesis

The association between the timing of vasopressor initiation and short-term mortality was synthesized using the Mantel-Haenszel method to calculate pooled odds ratios (ORs) with their 95% confidence intervals. In contrast, the association between the timing of vasopressor initiation and ICU length of stay was synthesized using Hedges’ g method and expressed as the standardized mean difference, with their 95% confidence intervals. If studies reported median and interquartile ranges for ICU length of stay, we transformed them into means and standard deviations using the method proposed by Wan et al. [11]. Heterogeneity among the included studies was assessed by I^2^ and χ^2^ tests. Random-effect models were used to pool effect estimates, given the nature of our study designs. To avoid potential bias in cohort studies, we synthesized the quantitative results from randomized trials and quasi-experimental studies as our primary results. Publication bias was assessed using Egger’s regression test. Meta-analyses were performed using the R package ‘meta’ (version 4.2.2). Statistical significance was set at a p-value of < 0.05.

### Grading quality of evidence

For assessing the quality of the evidence in this review, at least two independent reviewers (C. H. Ma, J. Healy, E. Kinteh, C. C. Ma) followed the Grading of Recommendations, Assessment, Development, and Evaluations (GRADE) guidelines to evaluate the quality of evidence for each outcome [12]. This approach categorizes the quality of evidence into four levels (high, moderate, low, and very low) based on the assessment of five factors: study design, risk of bias, inconsistency, indirectness, and imprecision.

In line with the latest guidance from the International Society for Pharmacoepidemiology [13], combining non-randomized trials with RCTs is permissible under certain conditions to avoid misleading and unreliable meta-analytical outcomes. We decided to combine two RCTs with quasi-experimental studies (QES) in our meta-analysis for three primary reasons as suggested by the guidance: (1) the presence of consistently relevant and applicable clinical conditions across study types ensuring comparability; (2) the limited availability of RCTs on the topic, with only two studies identified, indicating a gap in the literature; and (3) the implementation of rigorous statistical analysis in the non-randomized studies, specifically using propensity score matching methods, helps mitigate potential biases.

### Subgroup and sensitivity analysis

We observed different cut-off points for defining early and late groups, ranging from one to seven hours, as summarized in Table 1. To assess the impact of these varying cut-off points on short-term mortality, we divided the studies into two categories: (1) those with cut-off points between one and three hours and (2) those with cut-off points within one hour. A sensitivity analysis was conducted to evaluate the effects of these divisions.

In addition, we conducted two subgroup analyses to examine the influence of various factors on the outcomes when integrating quasi-experimental studies with randomized controlled trials in our findings: (1) the timing of vasopressor administration, defined as either the onset of septic shock or the time of the first fluid bolus, and (2) the use of norepinephrine monotherapy or use of additional vasopressors.

Based on the results of the risk of bias assessment, we conducted an additional sensitivity analysis by excluding lower-quality studies. Specifically, we removed article categorized as having “some concerns” for randomized controlled trial. This approach allowed us to assess the robustness of the findings by focusing on studies with a lower risk of bias.

**Table 1:** Study characteristics for articles included

Author, year	Study location	Predominant vasopressor	Study design	Number of patients		Definitions of timing administration of vasopressors		Mortality rate: early/late group (%)	Time zero	Composition of vasopressors
				Early	Late	Early (Median, IQR)	Late (Median, IQR)			
Bai, 2014 [4]	China	NE	Retrospective cohort study	86	127	Within 2 h	More than 2 h	28-day mortality: 29.1/43.3	Septic shock onset	NE monotherapy
Reardon, 2014 [14]	USA	AVP	Retrospective cohort study	35	36	Within 6 h	Between 6 and 48 h	Mortality: 88.6/88.9	Initiation of CA	Use of additional vasopressors
Elbouhy, 2019 [15]	Egypt	NE	Randomized controlled trial	57	44	25 (20–30) minutes	120 (120–180) minutes	In-hospital mortality: 28.1/54.5	Emergency room arrival	NE monotherapy
Permpikul, 2019 [16]	Thailand	NE	Randomized controlled trial	155	155	70 (50–90) minutes	167 (125–273) minutes	28-day mortality: 15.5/21.9	Emergency room arrival	Use of additional vasopressors
Hammond, 2019 [17]	USA	AVP	Retrospective cohort study	48	48	127.8 min	28.6 (5.3-187.6) hours	In-hospital mortality: 40/42	Septic shock onset	Use of additional vasopressors
Hidalgo, 2020 [5]	USA	NE	Retrospective cohort study	76	43	Within 6 h	More than 6 h	30-day mortality: 25/51.1	Septic shock onset	Use of additional vasopressors
Ospina-Tascón, 2020 [18]	Colombia	NE	Retrospective cohort study / quasi-experimental study	93	244	0 (0–60) minutes	180 (60–240) minutes	28-day mortality: 18.3/38.7	First fluid bolus	Use of additional vasopressors
Xu, 2022 [19]	USA	NE		2069	2184	Within 3 h	More than 3 h	28-day mortality: 30.0/37.8	Septic shock onset	Use of additional vasopressors
Yeo, 2022 [6]	Korea	NE		149	149	18 (6–42) minutes	138 (90–228) minutes	28-day mortality: 47.7/33.6	First fluid bolus	Use of additional vasopressors
Jouffroy, 2022 [20]	France	NE	Retrospective cohort study	143	355	Prehospital NE	Without prehospital NE	30-day mortality: 13.3/71.2	Septic shock onset	NE monotherapy
Rydz, 2022 [21]	USA	AVP	Retrospective cohort study	189	196	Within 7 h	More than 7 h	In-hospital mortality: 53.4/66.3	NE initiation	Use of additional vasopressors

*NE* norepinephrine, *AVP* vasopressin, *CA* catecholamine

## Results

### Search results

**Fig. 1:**
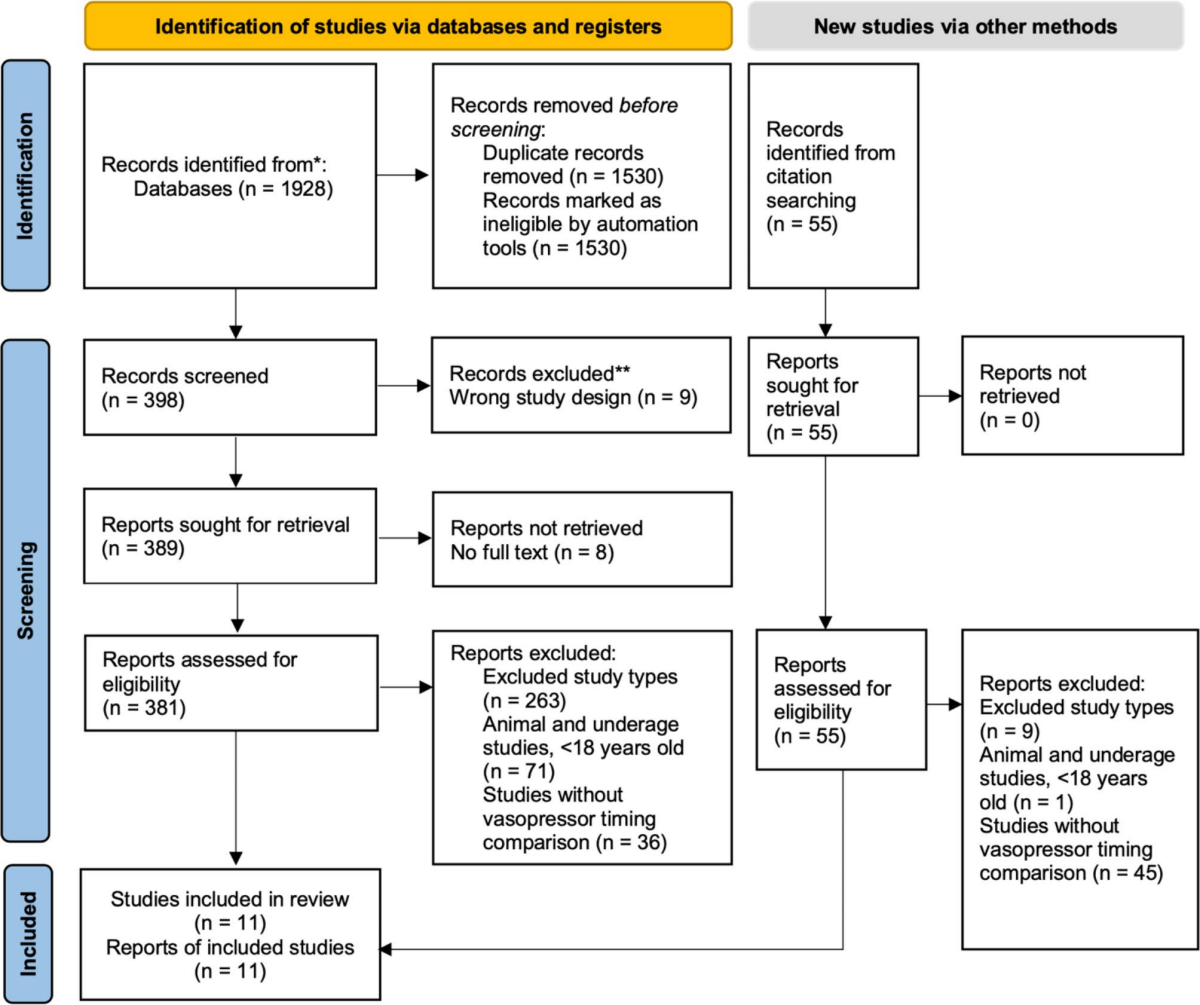
PRISMA flow diagram of the study selection process. * Consider, if feasible to do so, reporting the number of records identified from each database or register searched (rather than the total number across all databases/registers). ** If automation tools were used, indicate how many records were excluded by a human and how many were excluded by automation tools

A total of 398 articles were identified from the database search, with eleven included in the final analysis (Fig. 1). Among these studies, nine were cohort studies involving a total of 6,250 patients, and two were RCTs involving 411 patients (Table 1). Three of the nine cohort studies employed quasi-experimental designs employing propensity-score matching methods. Eight out of the eleven included studies used norepinephrine as the primary vasopressor, while three studies used vasopressin. In addition, only one RCT and two cohort studies assessed the effectiveness of norepinephrine monotherapy. Regarding fluid resuscitation, seven studies followed the guidelines recommended for early fluid expansion, using at least 30 mL/kg of crystalloid fluids. The infusion rate and volume of intravenous fluid therapy were determined by the treating clinician. Only one study described fluid resuscitation as administering repeated fluid challenges with crystalloids and/or 4% albumin, while another three studies did not specify their fluid resuscitation protocols.

### Primary outcome

**Fig. 2:**
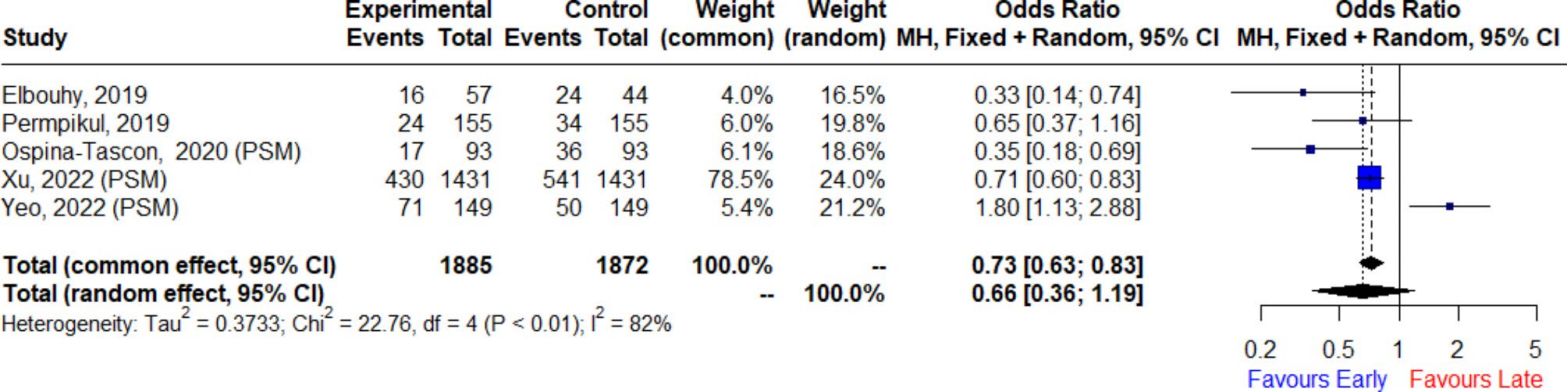
Forest plot pooling results from randomized controlled trials and quasi-experimental studies for short-term mortality

The risk of short-term mortality was significantly lower in the early vasopressor groups compared with the late groups in both cohort studies and RCTs (OR: 0.40, 95% CI: [0.21, 0.76], I^2^: 93% in cohorts vs.; OR: 0.49, 95% CI: [0.25, 0.96], I^2^: 45% in RCTs). However, there was no significant difference in short-term mortality based on quasi-experimental studies (OR: 0.78, 95% CI: [0.32, 1.90], I^2^: 89%). We focused on five studies in this analysis, all of which are either randomized controlled trials (RCTs) or quasi-experimental studies. These types of studies were chosen due to their higher methodological rigor and their ability to reduce bias when assessing the causal relationship between vasopressor timing and clinical outcomes. To evaluate the impact of combining quasi-experimental studies (QES) with RCTs on our findings, we employed a Bayesian three-level hierarchical model using the R package ‘brms’. This model allowed us to integrate the results from these two types of studies simultaneously, with the three levels corresponding to the study, group, and population levels (Table 2). Among 3,757 patients in the combined analysis of two RCTs and three quasi-experimental studies, we found no significant difference in short-term mortality between early and late administration of vasopressors (OR: 0.66, 95% CI: [0.36, 1.19], I^2^: 82%, Fig. 2). Further details regarding the comprehensive results of the primary outcome can be found in the supplementary information (Additional file 1: Figures S1, S2, S3, S4, S9, S10).

### Secondary outcome

**Fig. 3:**
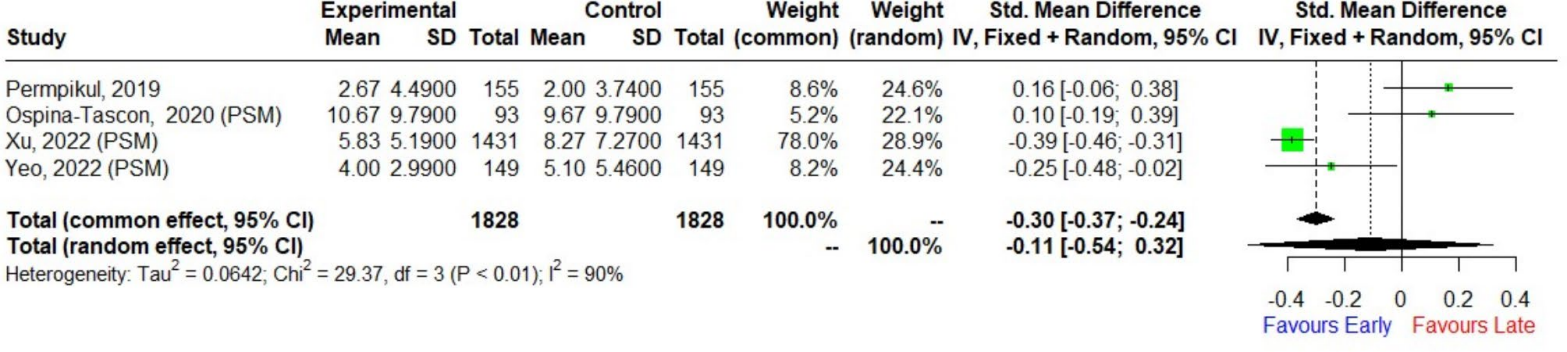
Forest plot pooling results from randomized controlled trials and quasi-experimental studies for ICU length of stay

One randomized controlled trial (RCT), seven cohort studies, and three quasi-experimental studies included data on ICU length of stay. We found no significant difference between the early and the late groups (SMD: 0.16, 95% CI: [-0.06, 0.38] in RCTs; SMD: -0.07, 95% CI: [-0.34, 0.20], I^2^: 94% in cohort studies; SMD: -0.20, 95% CI: [-0.82, 0.41], I^2^: 82% in quasi-experimental studies). In the combined analysis of one RCT and three quasi-experimental studies, there was no significant difference between the early and the late groups (SMD: -0.11, 95% CI: [-0.54, 0.32], I^2^: 90%, Fig. 3). Further details regarding the comprehensive results of secondary outcome can be found in supplementary information (Additional file 1: Figures S5, S6, S7, S8).

### Risk of bias assessment

In the risk of bias assessment of the two RCTs included in our study, one demonstrated a low risk in each domain, while the other exhibited a minor risk of bias due to the randomization process and reporting results (Fig. S17). For bias due to confounding, five cohorts and three quasi-experimental studies showed moderate risk, whereas the remaining two cohorts studies reported a serious risk, resulting in an overall serious risk assessment (Fig. S18).

### Subgroup and sensitivity analysis

**Fig. 4:**
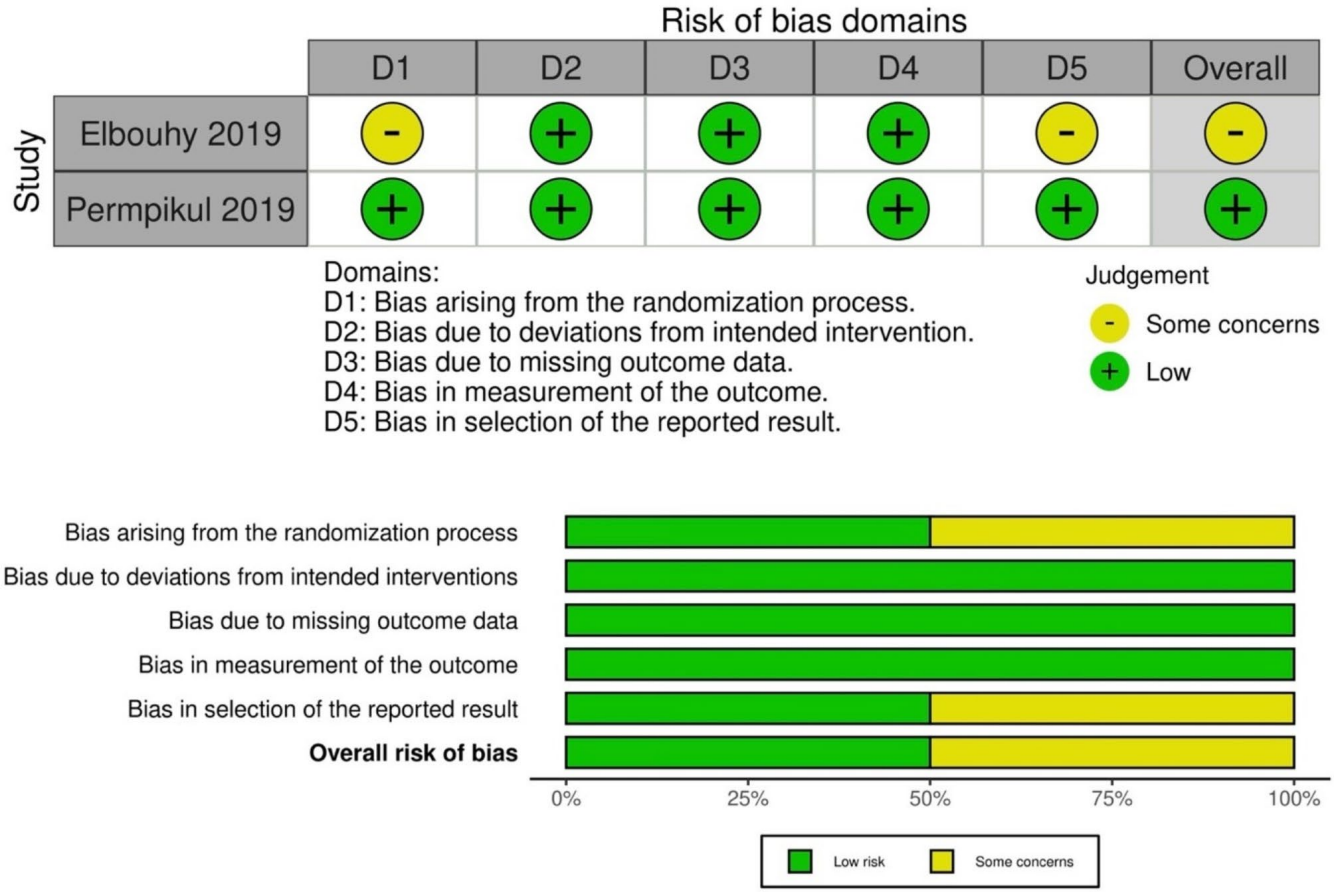
Sensitivity analysis of different cut-off points of early administration of vasopressors

In the analysis comparing different cut-off points for early versus late vasopressor administration, initiating vasopressors within one to three hours was associated with a significant reduction in short-term mortality, with high consistency across studies (OR: 0.70, 95% CI: [0.60, 0.82], I^2^: 0%, Fig. 4a). However, no significant difference in mortality was observed when comparing patients who received vasopressors within the first hour, referred to as the “extremely early” administration group, to those who received vasopressors later (OR: 0.61, 95% CI: [0.20, 1.89], I^2^: 91%, Fig. 4b). No significant difference was observed between early and late administration within the group administered medication within one hour, the “extremely early” administration subgroup (OR: 0.61, 95% CI: [0.20, 1.89], I^2^: 91%, Fig. 4b). In addition, Bayesian three-level hierarchical analysis of RCTs and quasi-experimental studies revealed no significant difference in short-term mortality between early and late groups (OR: 0.62, 95% CI: [0.32, 1.22]). Further details regarding the comprehensive results of subgroup and sensitivity analyses can be found in the supplementary information (Additional file 1: Figures S13, S14, S15, S16).

We also compared studies with different definitions of “time zero” for vasopressor administration. Early vasopressor administration was associated with a statistically significant reduction in short-term mortality in studies that defined “time zero” as the onset of septic shock (OR: 0.64, 95% CI: [0.48, 0.85], I^2^: 39%). Studies that defined “time zero” as the time of the first fluid bolus showed no statistical difference in short-term mortality between early versus late vasopressor imitation (OR: 0.81, 95% CI: [0.17, 4.01], I^2^: 93%). Second, we compared studies involving norepinephrine monotherapy with those that utilized additional vasopressors. In the group of patients receiving NE monotherapy, early vasopressor therapy was associated with a statistically significant decrease in short-term mortality (OR: 0.33, 95% CI: [0.14, 0.74], results from one RCT) compared to the group of using of additional vasopressors (OR: 0.75, 95% CI: [0.40, 1.42], I^2^: 84%, Table 2).

To account for potential bias, we performed a sensitivity analysis by excluding studies deemed to have a higher risk of bias, and early vasopressor administration was still associated with a significant reduction in short-term mortality (OR: 0.74, 95% CI: [0.65, 0.85], I^2^: 84%). This consistency across higher-quality studies reinforces the reliability of our findings.

**Table 2:** Subgroup and sensitivity analyses

Subgroup/sensitivity analysis	Groups	Study	Study design	Primary outcome: Odds ratio (95% C.I.)	I-square value
Bayesian three-level analysis of RCT and quasi-experimental studies		Elbouhy, 2019	RCT	0.62 (95% Cr.I. 0.32, 1.22)	
		Permpikul, 2019	RCT		
		Ospina-Tascón, 2020	QES		
		Xu, 2022	QES		
		Yeo, 2022	QES		
Cut-off point of early administration	One-to-three hours	Permpikul, 2019	RCT	0.70 (0.60, 0.82)	0%
		Xu, 2022	QES		
	Within one hour	Elbouhy, 2019	RCT	0.61 (0.20, 1.89)	91%
		Ospina-Tascón, 2020	QES		
		Yeo, 2022	QES		
Definition of time zero	Septic shock	Elbouhy, 2019	RCT	0.64 (0.48, 0.85)	39%
		Permpikul, 2019	RCT		
		Xu, 2022	QES		
	First fluid bolus	Ospina-Tascón, 2020	QES	0.81 (0.17, 4.01)	93%
		Yeo, 2022	QES		
Composition of vasopressors	NE monotherapy	Elbouhy, 2019	RCT	0.33 (0.14, 0.74)	
	Use of additional vasopressors	Permpikul, 2019	RCT	0.75 (0.40, 1.42)	84%
		Ospina-Tascón, 2020	QES		
		Xu, 2022	QES		
		Yeo, 2022	QES		

*C.I*. Confidence interval, *Cr.I.* Credible interval, *QES* quasi-experimental study

### Grading quality of evidence

We downgraded the level of certainty of evidence based on the assessment of five different domains: study design, risk of bias, inconsistency, indirectness, and imprecision. This downgrade was influenced by factors such as the I-square value, a measure of inconsistency indicating inconsistency, and the 95% confidence interval of the odds ratio, reflecting imprecision. The study design for analyzing short-term mortality included two RCTs and three quasi-experimental studies. Consequently, the level of certainty of the evidence was downgraded to moderate based on inconsistency and imprecision. The subgroup analysis of the cut-off point for early administration at one to three hours revealed I-square values below 50% and a 95% confidence interval of the odds ratio crossing the threshold of 0.75, indicating a high level of certainty of evidence. In contrast, the level of certainty of evidence for the other three studies, which set the cut-off point for extremely early administration within one hour, was downgraded to very low. Further details regarding the comprehensive grading quality of evidence can be found in the supplementary information (Additional file 1: Table S4, S5).

## Discussion

To our knowledge, this systematic review and meta-analysis represent the largest endeavor conducted to determine the optimal timing of early initiation of vasopressor therapy in adults with septic shock. In this study, we specifically combined results from two RCTs and three quasi-experimental studies with 3,757 patients to avoid possible selection bias. Overall, we found that early administration of vasopressors was not associated with short-term mortality. This finding was inconsistent with the previous meta-analysis published in 2020 by Li, et al. [8]. Their analysis encompassed five studies with 929 patients, including two RCTs and three cohort studies, with limitations of small sample sizes, inconsistent definitions of early and late groups, and the presence of selection bias and confounding variables. Using a more sophisticated statistical analysis to count for the issue of different study designs, our Bayesian three-level analysis with RCTs and quasi-experimental studies revealed no significant difference observed in short-term mortality between early and late vasopressor initiation.

However, we found that early, but not extremely early, administration of vasopressors was found to be associated with a statistically significant lower risk of short-term mortality, supported by high certainty of evidence. Specifically, administering vasopressors within one to three hours was associated with a statistically significant decrease in short-term mortality, whereas administration within the first hour did not yield significant improvements. According to the Surviving Sepsis Campaign Bundle update in 2018, the ‘hour-1 bundle’ has been recognized as a critical intervention in sepsis management [22]. This bundle includes measuring lactate, collecting blood cultures before initiating antibiotics, administering broad-spectrum antibiotics, and providing intravenous fluids and vasopressors—all within one hour of sepsis recognition. However, a multicenter retrospective cohort study published in 2021 found that the group that achieved the hour-1 bundle did not significantly improve in-hospital mortality. Conversely, hour-3 and hour-6 bundle achievements showed significantly lower odds ratios of in-hospital mortality [23]. Furthermore, another retrospective cohort study from 2018 did not show a significant difference in mortality between patients who received the hour-1 bundle and those who did not, even after adjusting for potential confounding variables [24]. These studies support our finding that extremely early administration of vasopressors within the first hour of septic shock may not improve patients’ clinical outcomes.

In managing septic shock, the timing of vasopressor administration and fluid resuscitation is a crucial consideration. It has been observed that the early use of vasopressors without adequate fluid resuscitation may exacerbate the reduction in tissue perfusion [25]. Ma et al. found that a greater amount of fluid infusion and appropriate dosing of norepinephrine were necessary for achieving a more favorable early clinical outcome, while less fluid infusion was found to be beneficial in the late phase. A multicenter prospective cohort study published in 2020 indicated that the effect of vasopressors on hemodynamics and outcome depends on the fluid status at the time of vasopressor administration [26]. As fluid volume increased, the association between vasopressor dosing intensity and increased mortality was attenuated. Our finding, which showed significant differences in short-term mortality within early rather than extremely early groups, emphasized the need for prioritizing adequate fluid infusion before the use of vasopressors. Further research is needed to investigate this relationship thoroughly.

We also observed a statistically significant reduction in short-term mortality with early administration of norepinephrine monotherapy compared to the use of additional vasopressors. Notably, a systematic review published in 2012 [27] and a meta-analysis published in 2011 [28] found norepinephrine to be superior to dopamine in terms of 28-day mortality, hemodynamic status, and reduced adverse events. However, since norepinephrine is recommended as the primary vasopressor in the ‘Surviving Sepsis Campaign Guidelines in 2021’, the use of a combination of vasopressors in observational studies may indicate a more severe patient condition and potentially worse outcomes. As our subgroup analysis included only one RCT focused on monotherapy, further studies are warranted to validate our findings.

Our study has important limitations. First, most of the included studies were observational and retrospective, which inherently introduces the potential for selection bias and confounding. However, our attempt to synthesize evidence from randomized control trials and quasi-experimental observational studies alleviates the potential bias. Additionally, the variability in the definitions used for “early” and “late” initiation of vasopressors among the included studies may introduce heterogeneity and limit direct comparisons. Another limitation is that not all patients in the included studies were admitted to the ICU. For example, in the study by Yeo et al., only 65% of patients were ultimately admitted to the ICU, despite all receiving vasopressors. Studies focused exclusively on ICU patients may yield different outcomes compared to those including patients from the emergency department, where treatment protocols vary. This heterogeneity between settings could influence the interpretation of the association between vasopressor timing and patient outcomes, potentially affecting the generalizability of our findings. In our subgroup analyses, we found studies using septic shock to define their “time zero” and using one to three hours as the cut-off points of early administration had relatively low heterogeneity and more reliable suggestions. We explored potential sources of heterogeneity through subgroup analyses and sensitivity analyses; however, the presence of heterogeneity may still influence the overall certainty and generalizability of our findings. In addition, one limitation is the inconsistent reporting of vasopressor-free days across studies, which impacts the relevance of the secondary outcome. Only three studies—Permpikul et al. 2019, Hidalgo et al. 2020, and Xu et al. 2022—provided explicit data on vasopressor-free days. The variation in reporting periods, such as 28 days versus 72 h, complicates direct comparison. Most other studies only mentioned the duration of vasopressor use without detailed reporting. This variability limits our ability to assess the impact of timing on vasopressor-free days and underscores the need for standardized outcome reporting in future research.

## Conclusion

In conclusion, our study suggests that extremely early initiation of vasopressor therapy does not decrease mortality in patients with septic shock. However, the distinction between “early” and “very early” initiation remains uncertain due to potential confounding factors and the heterogeneity in study definitions and adjustments. Future research should focus on conducting RCTs to confirm these findings and to further elucidate the biological mechanisms underlying the association between early vasopressor initiation and mortality.

## Electronic supplementary material

Below is the link to the electronic supplementary material.

Supplementary Material 1: Additional file 1: Table S1. Search strategy of PubMed. Table S2. Search strategy of Embase. Table S3. Search strategy of Cochrane. Figure S1. Forest plot pooling results from cohort studies (include quasi-experimental studies) and randomized controlled trials for short-term mortality. Figure S2. Forest plot pooling results from randomized controlled trials for short-term mortality. Figure S3. Forest plot pooling results from cohort studies (only include original) for short-term mortality. Figure S4. Forest plot pooling results from quasi-experimental studies for short-term mortality. Figure S5. Forest plot pooling results from cohort studies (include quasi-experimental studies) and randomized controlled trials for ICU length of stay. Figure S6. Forest plot pooling results from randomized controlled trials for ICU length of stay. Figure S7. Forest plot pooling results from cohort studies (only include original) for ICU length of stay. Figure S8. Forest plot pooling results from quasi-experimental studies for ICU length of stay. Figure S9. Meta-analysis result based on cohort studies (include quasi-experimental studies) and randomized controlled trials. Figure S10. Funnel plots of cohort studies (left) and randomized controlled trials (right). Figure S11. Forest plot pooling results from cohort studies (include quasi-experimental studies) and randomized controlled trials that exclude studies on vasopressin. Figure S12. Forest plot pooling results from cohort studies (include quasi-experimental studies) and randomized controlled trials that exclude studies with high risk of bias. Figure S13. Forest plot pooling results from randomized controlled trials and quasi-experimental studies that defined “time zero” as the onset of septic shock for short-term mortality. Figure S14. Forest plot pooling results from randomized controlled trials and quasi-experimental studies that defined “time zero” as the first fluid bolus for short-term mortality. Figure S15. Forest plot pooling results from randomized controlled trials and quasi-experimental studies that used additional vasopressors for short-term mortality. Figure S16. Forest plot pooling results from randomized controlled trials and quasi-experimental studies that excluded lower-quality study. Table S4. Grading quality of evidence for short-term mortality and ICU length of stay. Table S5. Grading quality of evidence for short-term mortality of subgroup and sensitivity analyses. Figure S17. Risk of bias of randomized controlled trials. Figure S18. Risk of bias of observational studies

Supplementary Material 2

## Data Availability

The full dataset is freely available online on GitHub (https://github.com/wujinja-cgu/data/blob/main/Data-Extremely%20Early%20Initiation%20of%20Vasopressors%20Might%20not%20Decrease%20Short-Term%20Mortality%20for%20Adults%20with%20Septic%20Shock.xlsx).
