# Supplementary material for: Extremely early initiation of vasopressors might not decrease short-term mortality for adults with septic shock: a systematic review and meta-analysis
Source: Ann Intensive Care. 2025 Jan 27;15:18. doi: 10.1186/s13613-025-01428-0 (PMC11769887; doi:10.1186/s13613-025-01428-0)

**Supplemental Materials**

**Extremely Early Initiation of Vasopressors Might not Decrease Short-Term Mortality for Adults with Septic Shock: A Systematic Review and Meta-Analysis**

Cheng-Hsin Ma, Jack Healy, Ebrima Kinteh, Cheng-Chin Ma, Ching-Fang Tiffany Tzeng, Eric, H. Chou, Chin-Chieh Wu, Kuan-Fu Chen

Contents

Appendix 1. Search strategy3

Decision of study period3

Table S1. Search strategy of PubMed4

Table S2. Search strategy of Embase6

Table S3. Search strategy of Cochrane6

Appendix 2. Primary outcome and secondary outcome7

Figure S1. Forest plot pooling results from cohort studies (include quasi-experimental studies) and randomized controlled trials for short-term mortality7

Figure S2. Forest plot pooling results from randomized controlled trials for short-term mortality7

Figure S3. Forest plot pooling results from cohort studies (only include original) for short-term mortality7

Figure S4. Forest plot pooling results from quasi-experimental studies for short-term mortality7

Figure S5. Forest plot pooling results from cohort studies (include quasi-experimental studies) and randomized controlled trials for ICU length of stay8

Figure S6. Forest plot pooling results from randomized controlled trials for ICU length of stay8

Figure S7. Forest plot pooling results from cohort studies (only include original) for ICU length of stay8

Figure S8. Forest plot pooling results from quasi-experimental studies for ICU length of stay8

Figure S9. Meta-analysis result based on cohort studies (include quasi-experimental studies) and randomized controlled trials9

Figure S10. Funnel plots of cohort studies (left) and randomized controlled trials (right)9

Appendix 3. Different levels of analysis10

Analysis excluding studies on vasopressin10

Figure S11. Forest plot pooling results from cohort studies (include quasi-experimental studies) and randomized controlled trials that exclude studies on vasopressin10

Analysis excluding studies with high risk of bias10

Figure S12. Forest plot pooling results from cohort studies (include quasi-experimental studies) and randomized controlled trials that exclude studies with high risk of bias10

Appendix 4. Subgroup and sensitivity analyses 11

Figure S13. Forest plot pooling results from randomized controlled trials and quasi-experimental studies that defined “time zero” as the onset of septic shock for short-term mortality11

Figure S14. Forest plot pooling results from randomized controlled trials and quasi-experimental studies that defined “time zero” as the first fluid bolus for short-term mortality11

Figure S15. Forest plot pooling results from randomized controlled trials and quasi-experimental studies that used additional vasopressors for short-term mortality11

Figure S16. Forest plot pooling results from randomized controlled trials and quasi-experimental studies that excluded lower-quality study11

Appendix 5. Grading quality of evidence12

Table S4. Grading quality of evidence for short-term mortality and ICU length of stay12

Table S5. Grading quality of evidence for short-term mortality of subgroup and sensitivity analyses13

Appendix 6. Risk of bias assessment14

Figure S17. Risk of bias of randomized controlled trials14

Figure S18. Risk of bias of observational studies14

**Appendix 1. Search strategy**

**Decision of study period**

The decision to begin the study period in January 2010 was made with careful consideration of several factors. While no major changes in vasopressor treatment protocols prompted the choice of this specific starting point, focusing on studies from the last decade allows for the inclusion of data that more accurately reflects current clinical practices. Including studies much older than ten years might introduce findings that are less aligned with modern treatment standards, given the continuous advancements in sepsis management. Starting in 2010 strikes a balance between ensuring relevance and gathering sufficient data for robust analysis.

**Table S1.** Search strategy of PubMed

| **Search number** | **Query** | **Search Details** | **Results** |
| --- | --- | --- | --- |
| 1 | Septic Shock | "shock, septic"[MeSH Terms] OR ("shock"[All Fields] AND "septic"[All Fields]) OR "septic shock"[All Fields] OR ("septic"[All Fields] AND "shock"[All Fields]) | 43,152 |
| 2 | Vasopressor | "vasoconstrictor agents"[Pharmacological Action] OR "vasoconstrictor agents"[MeSH Terms] OR ("vasoconstrictor"[All Fields] AND "agents"[All Fields]) OR "vasoconstrictor agents"[All Fields] OR "vasopressor"[All Fields] OR "vasopressors"[All Fields] | 284,516 |
| 3 | Norepinephrine | "noradrenaline s"[All Fields] OR "norepinephrin"[All Fields] OR "norepinephrine"[MeSH Terms] OR "norepinephrine"[All Fields] OR "noradrenalin"[All Fields] OR "noradrenaline"[All Fields] OR "norepinephrines"[All Fields] | 131,189 |
| 4 | #2 OR #3 | "vasoconstrictor agents"[Pharmacological Action] OR "vasoconstrictor agents"[MeSH Terms] OR ("vasoconstrictor"[All Fields] AND "agents"[All Fields]) OR "vasoconstrictor agents"[All Fields] OR "vasopressor"[All Fields] OR "vasopressors"[All Fields] OR ("noradrenaline s"[All Fields] OR "norepinephrin"[All Fields] OR "norepinephrine"[MeSH Terms] OR "norepinephrine"[All Fields] OR "noradrenalin"[All Fields] OR "noradrenaline"[All Fields] OR "norepinephrines"[All Fields]) | 323,640 |
| 5 | Time | "time"[MeSH Terms] OR "time"[All Fields] | 5,169,396 |
| 6 | Timing | "timely"[All Fields] OR "timing"[All Fields] OR "timings"[All Fields] | 276,684 |
| 7 | Initiation | "initial"[All Fields] OR "initially"[All Fields] OR "initials"[All Fields] OR "initiate"[All Fields] OR "initiated"[All Fields] OR "initiates"[All Fields] OR "initiating"[All Fields] OR "initiation"[All Fields] OR "initiations"[All Fields] OR "initiator"[All Fields] OR "initiators"[All Fields] | 1,819,487 |

**Table S1.** Search strategy of PubMed

| **Search number** | **Query** | **Search Details** | **Results** |
| --- | --- | --- | --- |
| 8 | #5 OR #6 OR #7 | "time"[MeSH Terms] OR "time"[All Fields] OR "timely"[All Fields] OR "timing"[All Fields] OR "timings"[All Fields] OR "initial"[All Fields] OR "initially"[All Fields] OR "initials"[All Fields] OR "initiate"[All Fields] OR "initiated"[All Fields] OR "initiates"[All Fields] OR "initiating"[All Fields] OR "initiation"[All Fields] OR "initiations"[All Fields] OR "initiator"[All Fields] OR "initiators"[All Fields] | 6,685,802 |
| 9 | Adult: 19+ years, Young Adult: 19-24 years, Adult: 19-44 years, Middle Aged + Aged: 45+ years, Middle Aged: 45-64 years, Aged: 65+ years, 80 and over: 80+ years, from 2010/1/1 - 2023/11/30 | (("shock, septic"[MeSH Terms] OR ("shock"[All Fields] AND "septic"[All Fields]) OR "septic shock"[All Fields] OR ("septic"[All Fields] AND "shock"[All Fields])) AND ("vasoconstrictor agents"[Pharmacological Action] OR "vasoconstrictor agents"[MeSH Terms] OR ("vasoconstrictor"[All Fields] AND "agents"[All Fields]) OR "vasoconstrictor agents"[All Fields] OR "vasopressor"[All Fields] OR "vasopressors"[All Fields] OR ("noradrenaline s"[All Fields] OR "norepinephrin"[All Fields] OR "norepinephrine"[MeSH Terms] OR "norepinephrine"[All Fields] OR "noradrenalin"[All Fields] OR "noradrenaline"[All Fields] OR "norepinephrines"[All Fields])) AND ("time"[MeSH Terms] OR "time"[All Fields] OR ("timely"[All Fields] OR "timing"[All Fields] OR "timings"[All Fields]) OR ("initial"[All Fields] OR "initially"[All Fields] OR "initials"[All Fields] OR "initiate"[All Fields] OR "initiated"[All Fields] OR "initiates"[All Fields] OR "initiating"[All Fields] OR "initiation"[All Fields] OR "initiations"[All Fields] OR "initiator"[All Fields] OR "initiators"[All Fields]))) AND ((2010/1/1:3000/12/12[pdat]) AND (alladult[Filter] OR youngadult[Filter] OR adult[Filter] OR middleagedaged[Filter] OR middleaged[Filter] OR aged[Filter] OR 80andover[Filter])) | 422 |

**Table S2.** Search strategy of Embase

| **Search number** | **Search Details** | **Results** |
| --- | --- | --- |
| 1 | ('septic shock'/exp/mj OR 'sepsis-associated hypotension' OR 'septic shock' OR 'septicaemic shock' OR 'septicemic shock' OR 'shock, septic') AND ('hypertensive agent'/exp/mj OR 'antihypotensive agent' OR 'antihypotonic agent' OR 'hypertensive agent' OR 'hypertensive drug' OR 'pressor agent' OR 'vasopressor agent' OR 'vasopressor drug' OR vasopressor OR 'noradrenalin'/exp/mj OR norepinephrine) AND ('time'/exp/mj OR 'time' OR 'timing'/exp/mj OR 'initiation'/exp/mj) | 1398 |
| 2 | #1 AND (2010:py OR 2011:py OR 2012:py OR 2013:py OR 2014:py OR 2015:py OR 2016:py OR 2017:py OR 2018:py OR 2019:py OR 2020:py OR 2021:py OR 2022:py OR 2023:py) AND ([adult]/lim OR [aged]/lim OR [middle aged]/lim OR [very elderly]/lim OR [young adult]/lim) | 797 |

**Table S3.** Search strategy of Cochrane

| **Search number** | **Search Details** | **Results** |
| --- | --- | --- |
| 1 | MeSH descriptor: [Shock, Septic] explode all trees | 1401 |
| 2 | (septic shock):ti,ab,kw | 4052 |
| 3 | #1 OR #2 | 4052 |
| 4 | MeSH descriptor: [Vasoconstrictor Agents] explode all trees | 2272 |
| 5 | (Vasoconstrictor Agents):ti,ab,kw | 2152 |
| 6 | #4 OR #5 | 2463 |
| 7 | (vasopressor):ti,ab,kw | 2350 |
| 8 | MeSH descriptor: [Norepinephrine] explode all trees | 3479 |
| 9 | (norepinephrine):ti,ab,kw | 7554 |
| 10 | #8 OR #9 | 7606 |
| 11 | #6 OR #7 OR #10 | 11324 |
| 12 | MeSH descriptor: [Time] explode all trees | 88073 |
| 13 | (time):ti,ab,kw | 521067 |
| 14 | #12 OR #13 | 524470 |
| 15 | (timing):ti,ab,kw | 15211 |
| 16 | (initiation):ti,ab,kw | 32328 |
| 17 | #14 OR #15 OR #16 | 549866 |
| 18 | (adult):ti,ab,kw | 797916 |
| 19 | MeSH descriptor: [Adult] explode all trees | 612807 |
| 20 | #18 OR #19 | 925679 |
| 21 | #3 AND #11 AND #17 AND #20 | 201 |
| 22 | Filter with 2010/01/01-2023/11/30 | 172 |

**Appendix 2. Primary outcome and secondary outcome**

**1**

**Figure S2.** Forest plot pooling results from randomized controlled trials for short-term mortality
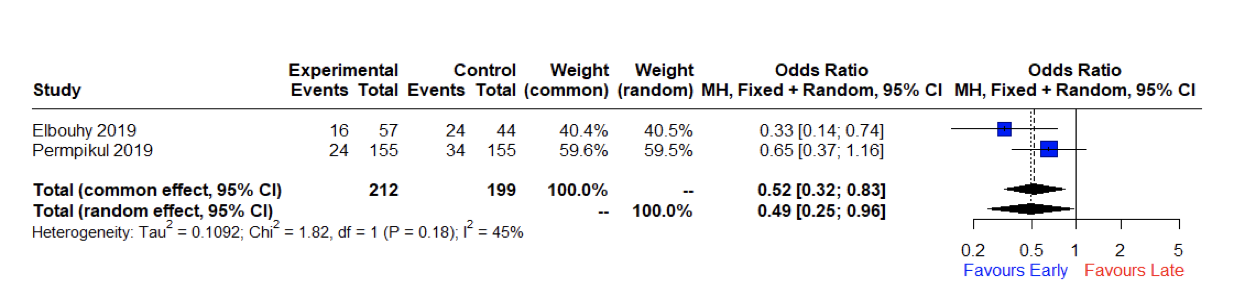


**Figure S3.** Forest plot pooling results from cohort studies (only include original) for short-term mortality


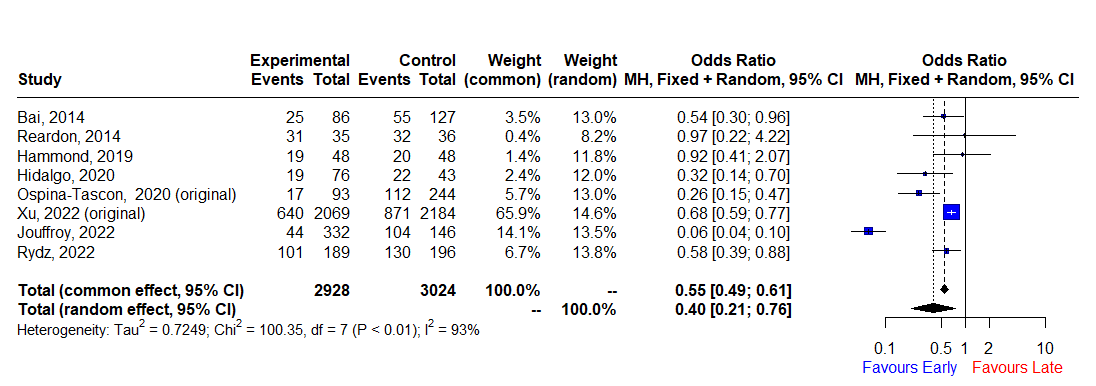


**Figure S4.** Forest plot pooling results from quasi-experimental studies for short-term mortality


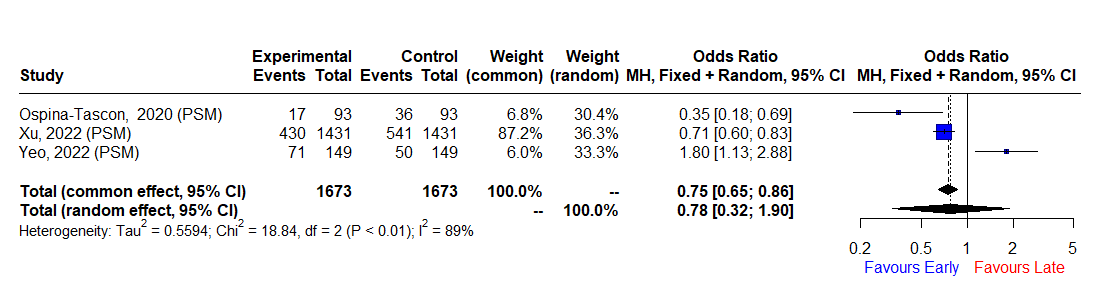


**5**

**Figure S6.** Forest plot pooling results from randomized controlled trials for ICU length of stay


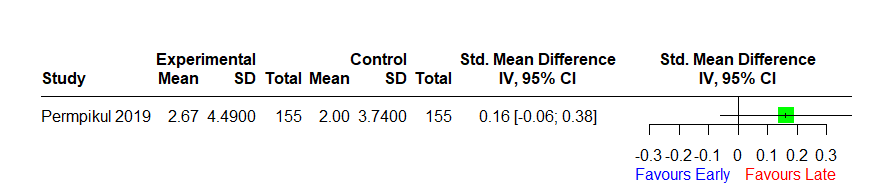


**Figure S7.** Forest plot pooling results from cohort studies (only include original) for ICU length of stay


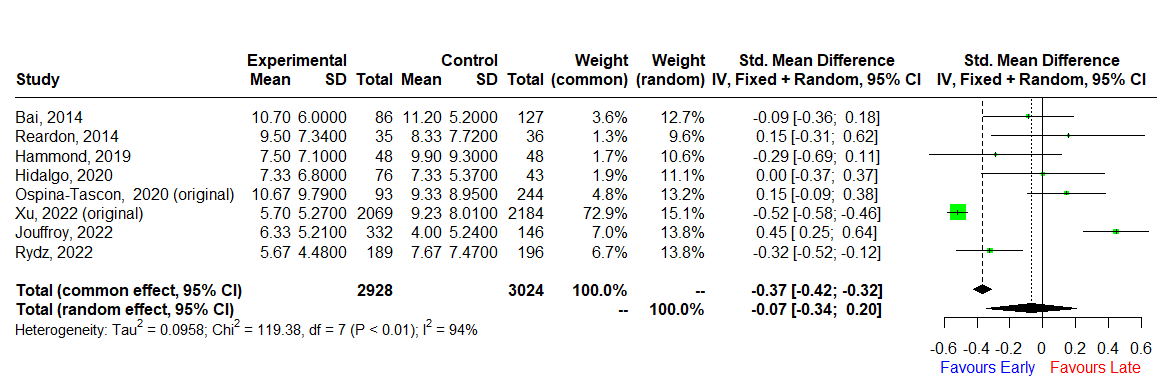


**Figure S8.** Forest plot pooling results from quasi-experimental studies for ICU length of stay


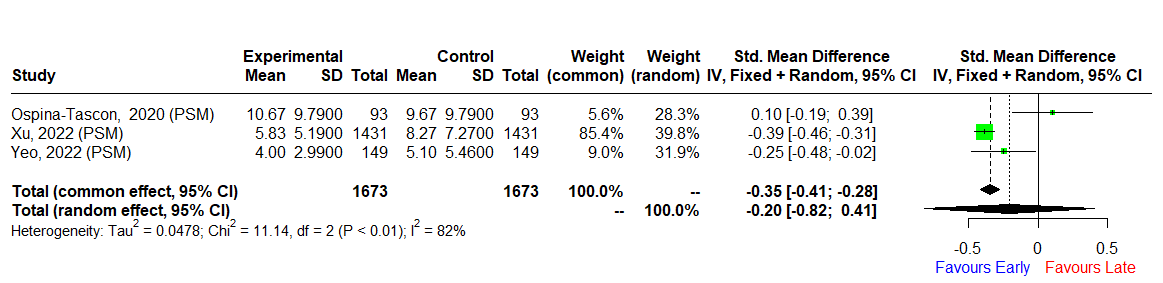


**Figure S9.** Meta-analysis result based on cohort studies (include quasi-experimental studies) and randomized controlled trials


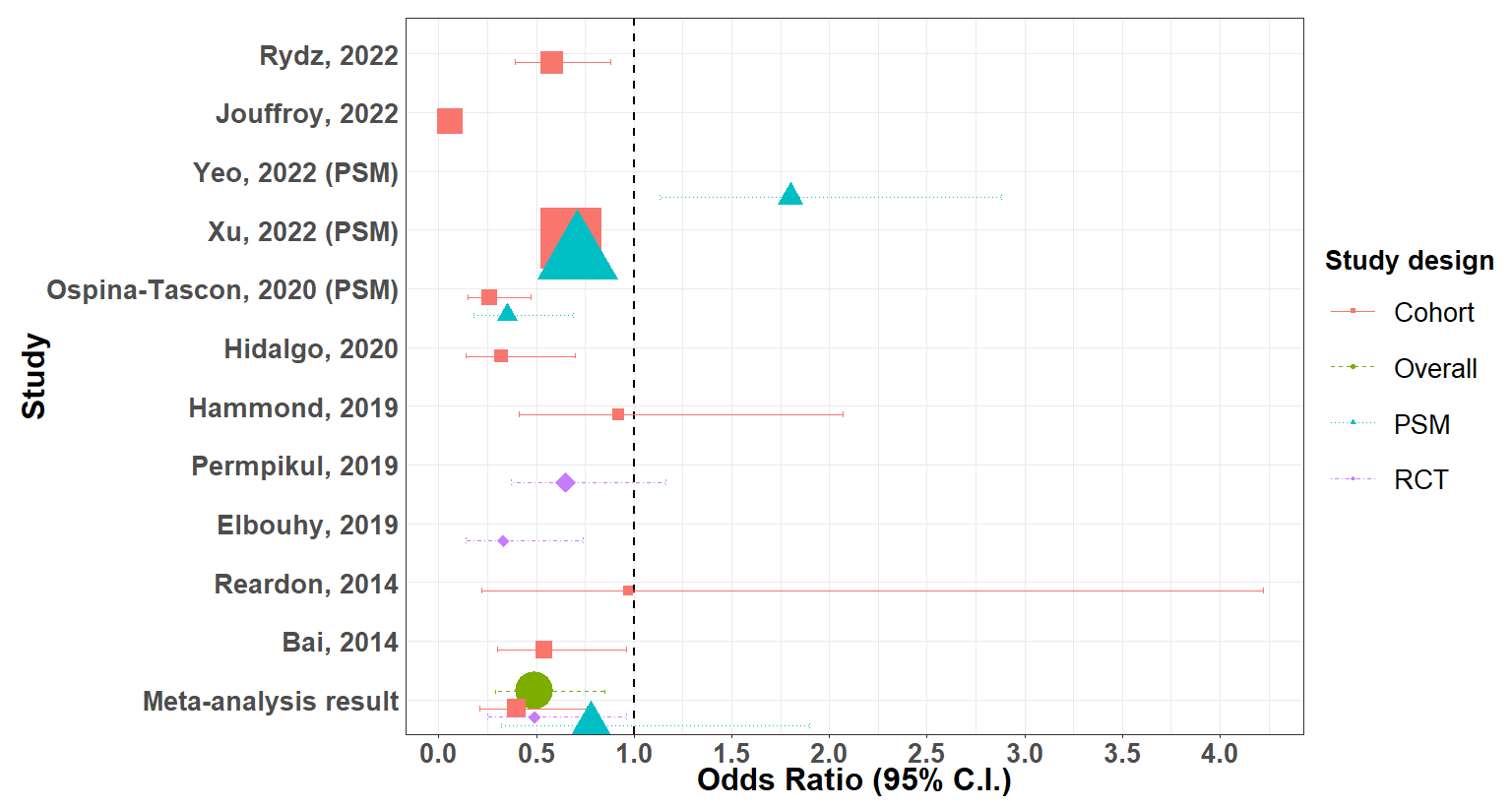


**Figure S10.** Funnel plots of cohort studies (left) and randomized controlled trials (right)

**
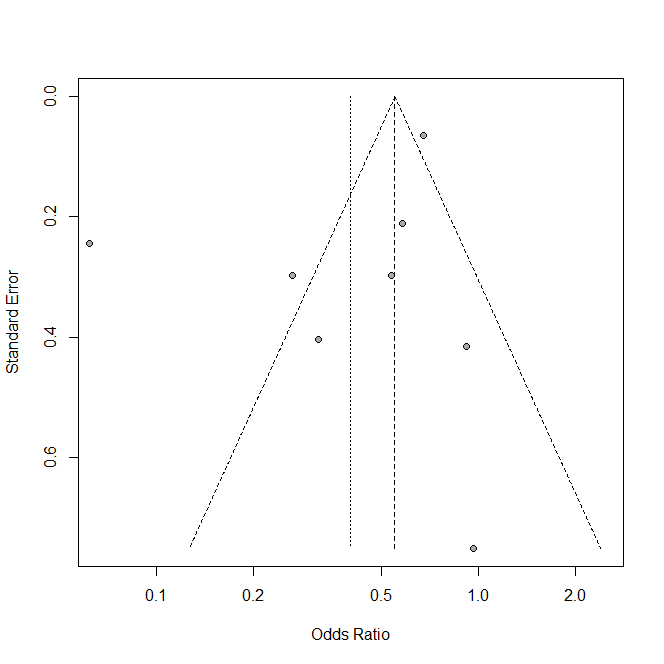

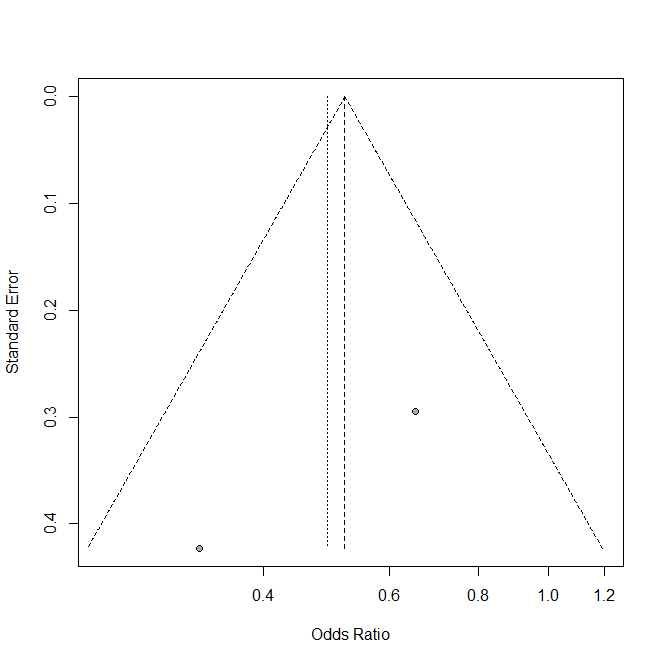
**

**Appendix 3. Different levels of analysis**

**Analysis excluding studies on vasopressin**

After excluding three studies where vasopressin was used as the primary vasopressor, in accordance with the "Surviving Sepsis Campaign: International Guidelines for the Management of Sepsis and Septic Shock 2021," the sensitivity analysis demonstrated a significant reduction in short-term mortality associated with early vasopressor administration (OR: 0.58, 95% CI: [0.51, 0.65], I^2^: 94%) (Figure. S11). This result indicates a robust association between early initiation of recommended vasopressors and improved outcomes, despite high heterogeneity across studies.

**Figure S11.** Forest plot pooling results from cohort studies (include quasi-experimental studies) and randomized controlled trials that exclude studies on vasopressin


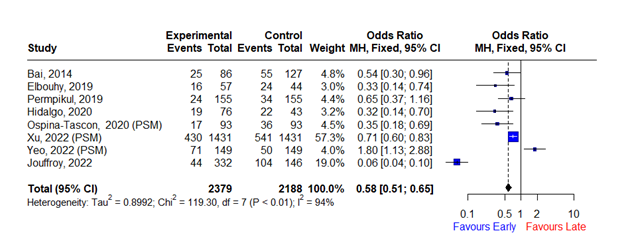


**Analysis excluding studies with high risk of bias**

To account for potential bias, we performed a sensitivity analysis by excluding studies deemed to have a higher risk of bias—those labeled as having "some concerns" for randomized controlled trials and "serious concerns" for observational studies. Despite these exclusions, early vasopressor administration was still associated with a significant reduction in short-term mortality (OR: 0.59, 95% CI: [0.52, 0.66], I^2^: 94%) (Figure S12). This consistency across higher-quality studies reinforces the reliability of our findings.

**Figure S12.** Forest plot pooling results from cohort studies (include quasi-experimental studies) and randomized controlled trials that exclude studies with high risk of bias


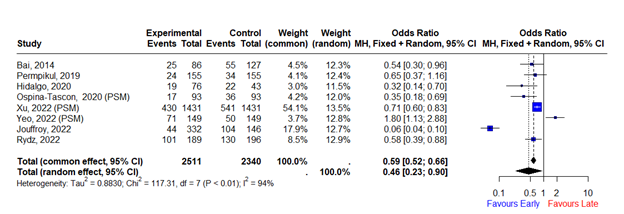


**Appendix 4. Subgroup and sensitivity analyses**

**Figure S13.** Forest plot pooling results from randomized controlled trials and quasi-experimental studies that defined “time zero” as the onset of septic shock for short-term mortality


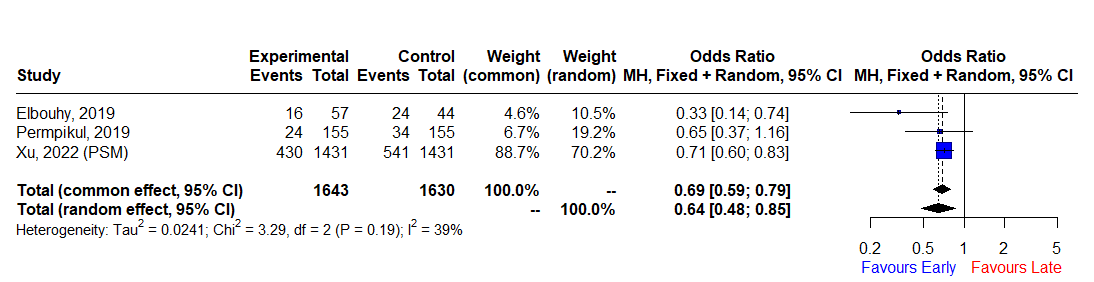


**Figure S14.** Forest plot pooling results from randomized controlled trials and quasi-experimental studies that defined “time zero” as the first fluid bolus for short-term mortality


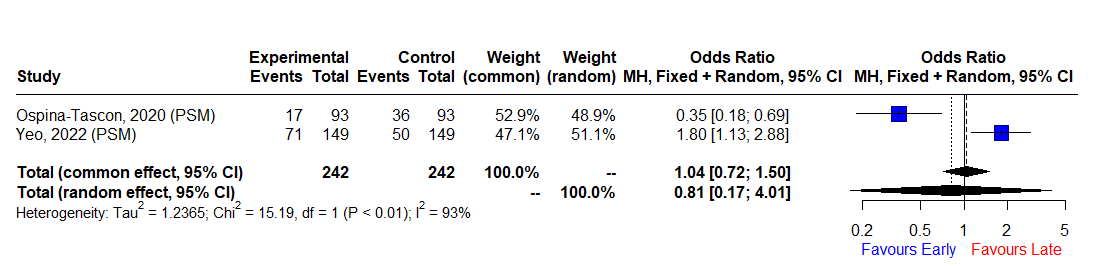


**Figure S15.** Forest plot pooling results from randomized controlled trials and quasi-experimental studies that used additional vasopressors for short-term mortality


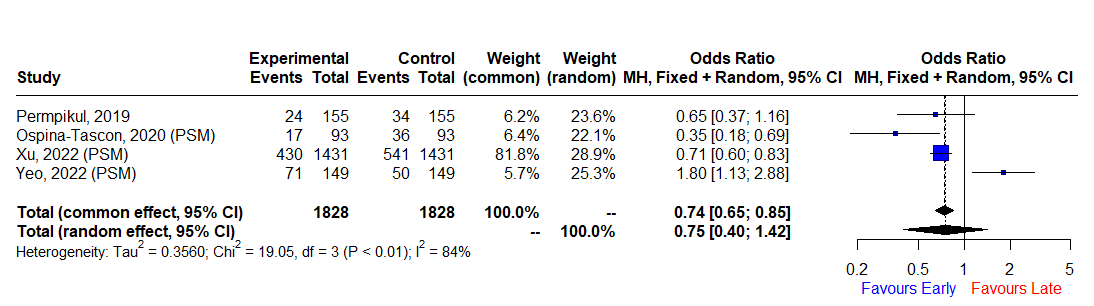


**Figure S16.** Forest plot pooling results from randomized controlled trials and quasi-experimental studies that excluded lower-quality study


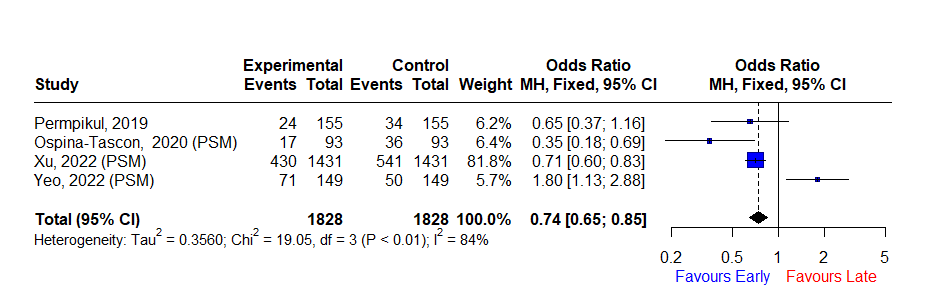


**Appendix 5.** Grading quality of evidence

**Table S4.** Grading quality of evidence for short-term mortality and ICU length of stay

| Certainty assessment | | | | | | Summary of findings | | | | | Importance |
| --- | --- | --- | --- | --- | --- | --- | --- | --- | --- | --- | --- |
| No. of studies | Study design | Risk of bias | Inconsistency | Indirectness | Imprecision | No. of patients | | Effect (95% CI) | | Certainty |  |
|  |  |  |  |  |  | Early | Late | Relative | Absolute |  |  |
| Mortality – randomized controlled trials | | | | | | | | | | | |
| 2 | RCT | not serious | not serious | not serious | serious ^a^ | 40/212 (18.9%) | 58/199 (29.1%) | OR 0.49 (0.25 to 0.96) | 124 fewer per 1,000 (from 198 fewer to 8 fewer) | ⊕⊕⊕⊕  High | CRITICAL |
| Mortality – cohort studies (only include original studies) | | | | | | | | | | | |
| 8 | observational studies | not serious | serious ^b, c^ | not serious | serious ^a^ | 896/2928 (30.6%) | 1346/3024 (44.5%) | OR 0.40 (0.21 to 0.76) | 202 fewer per 1,000 (from 301 fewer to 66 fewer) | ⊕⊕⊕⭘  Moderate | CRITICAL |
| Mortality – quasi-experimental studies | | | | | | | | | | | |
| 3 | QES | not serious | very serious ^b^ | not serious | very serious ^d^ | 518/1673 (31.0%) | 627/1673 (37.5%) | OR 0.78 (0.32 to 1.90) | 56 fewer per 1,000 (from 214 fewer to 158 more) | ⊕⭘⭘⭘  Very low | CRITICAL |
| ICU length of stay - randomized controlled trials | | | | | | | | | | | |
| 2 | RCT | not serious | not serious | not serious | serious ^e^ | 2.67 | 2 | - | SMD 0.16 SD higher (0.06 lower to 0.38 higher) | ⊕⊕⊕⊕  High | CRITICAL |
| ICU length of stay - cohort studies (only include original studies) | | | | | | | | | | | |
| 8 | observational studies | not serious | very serious ^b^ | not serious | serious ^e^ | 7.93 | 8.37 | - | SMD 0.07 SD lower (0.34 lower to 0.2 higher) | ⊕⊕⭘⭘  Low | CRITICAL |
| ICU length of stay - quasi-experimental studies | | | | | | | | | | | |
| 3 | QES | not serious | serious ^f^ | not serious | extremely  serious ^g^ | 5.94 | 8.07 | - | SMD 0.2 SD lower (0.82 lower to 0.41 higher) | ⊕⭘⭘⭘  Very low | CRITICAL |
| *CI* confidence interval, *OR* odds ratio, *SMD* standardized mean difference, *RCT* randomized controlled trials, *QES* quasi-experimental study  **Explanations**  a. OR 95% CI cross 0.75 without 1.25  b. I-square >85%  c. All favor early, so only rate down one level  d. OR 95% CI cross both 0.75 and 1.25  e. SMD 95% CI cross 0  f. 50%< I-square <85%  g. SMD 95% CI cross -0.8 and 0.4 | | | | | | | | | | | |

**Table S5.** Grading quality of evidence for short-term mortality of subgroup and sensitivity analyses

| Certainty assessment | | | | | | Summary of findings | | | | | Importance |
| --- | --- | --- | --- | --- | --- | --- | --- | --- | --- | --- | --- |
| No. of studies | Study design | Risk of bias | Inconsistency | Indirectness | Imprecision | No. of patients | | Effect (95% CI) | | Certainty |  |
|  |  |  |  |  |  | Early | Late | Relative | Absolute |  |  |
| Mortality – randomized controlled trials and quasi-experimental studies | | | | | | | | | | | |
| 5 | RCT and QES | not serious | serious ^a^ | not serious | serious ^b^ | 558/1885 (29.6%) | 685/1872 (36.6%) | OR 0.66 (0.36 to 1.19) | 90 fewer per 1,000 (from 194 fewer to 41 more) | ⊕⊕⊕⭘  Moderate | CRITICAL |
| Mortality – cut-off point set as one-to-three hours | | | | | | | | | | | |
| 2 | RCT and QES | not serious | not serious | not serious | serious ^b^ | 454/1586 (28.6%) | 575/1586 (36.3%) | OR 0.70 (0.60 to 0.82) | 78 fewer per 1,000 (from 108 fewer to 45 fewer) | ⊕⊕⊕⊕  High | CRITICAL |
| Mortality – cut-off point set as within one hour | | | | | | | | | | | |
| 3 | RCT and QES | not serious | very serious ^c^ | not serious | very serious ^d^ | 104/299 (34.8%) | 110/286 (38.5%) | OR 0.61 (0.20 to 1.89) | 109 fewer per 1,000 (from 274 fewer to 157 more) | ⊕⭘⭘⭘  Very low | CRITICAL |
| Mortality – “time zero” defined as septic shock onset | | | | | | | | | | | |
| 3 | RCT and QES | not serious | not serious | not serious | serious ^b^ | 470/1643 (28.6%) | 599/1630 (36.7%) | OR 0.64 (0.48 to 0.85) | 96 fewer per 1,000 (from 149 fewer to 37 fewer) | ⊕⊕⊕⊕  High | CRITICAL |
| Mortality – “time zero” defined as first fluid | | | | | | | | | | | |
| 2 | QES | not serious | very serious ^c^ | not serious | very serious ^d^ | 88/242 (36.4%) | 86/242 (35.5%) | OR 0.81 (0.17 to 4.01) | 47 fewer per 1,000 (from 270 fewer to 333 more) | ⊕⭘⭘⭘  Very low | CRITICAL |
| Mortality – Norepinephrine monotherapy | | | | | | | | | | | |
| 1 | RCT | not serious | not serious | not serious | not serious | 16/57 (28.1%) | 24/44 (54.5%) | OR 0.33 (0.14 to 0.74) | 262 fewer per 1,000 (from 402 fewer to 75 fewer) | ⊕⊕⊕⊕  High | CRITICAL |
| Mortality – use of additional vasopressor | | | | | | | | | | | |
| 2 | RCT and QES | not serious | serious ^a^ | not serious | very serious ^d^ | 542/1828 (29.6%) | 661/1828 (36.2%) | OR 0.75 (0.40 to 1.42) | 63 fewer per 1,000 (from 177 fewer to 84 more) | ⊕⊕⭘⭘  Low | CRITICAL |
| *CI* confidence interval, *OR* odds ratio, *RCT* randomized controlled trials, *QES* quasi-experimental study  **Explanations**  a. 50%< I-square <85%  b. OR 95% CI cross 0.75 without 1.25  c. I-square >85%  d. OR 95% CI cross both 0.75 and 1.25 | | | | | | | | | | | |

**Appendix 6.** Risk of bias assessment

**Figure S17.** Risk of bias of randomized controlled trials


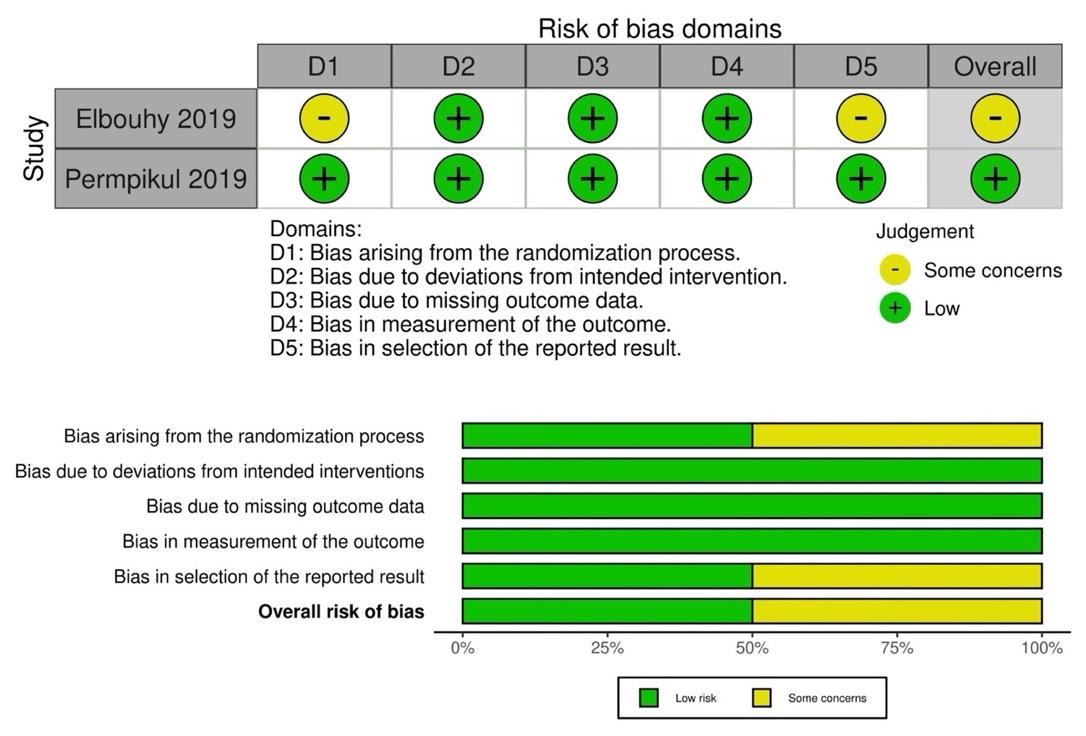


**Figure S18.** Risk of bias of observational studies


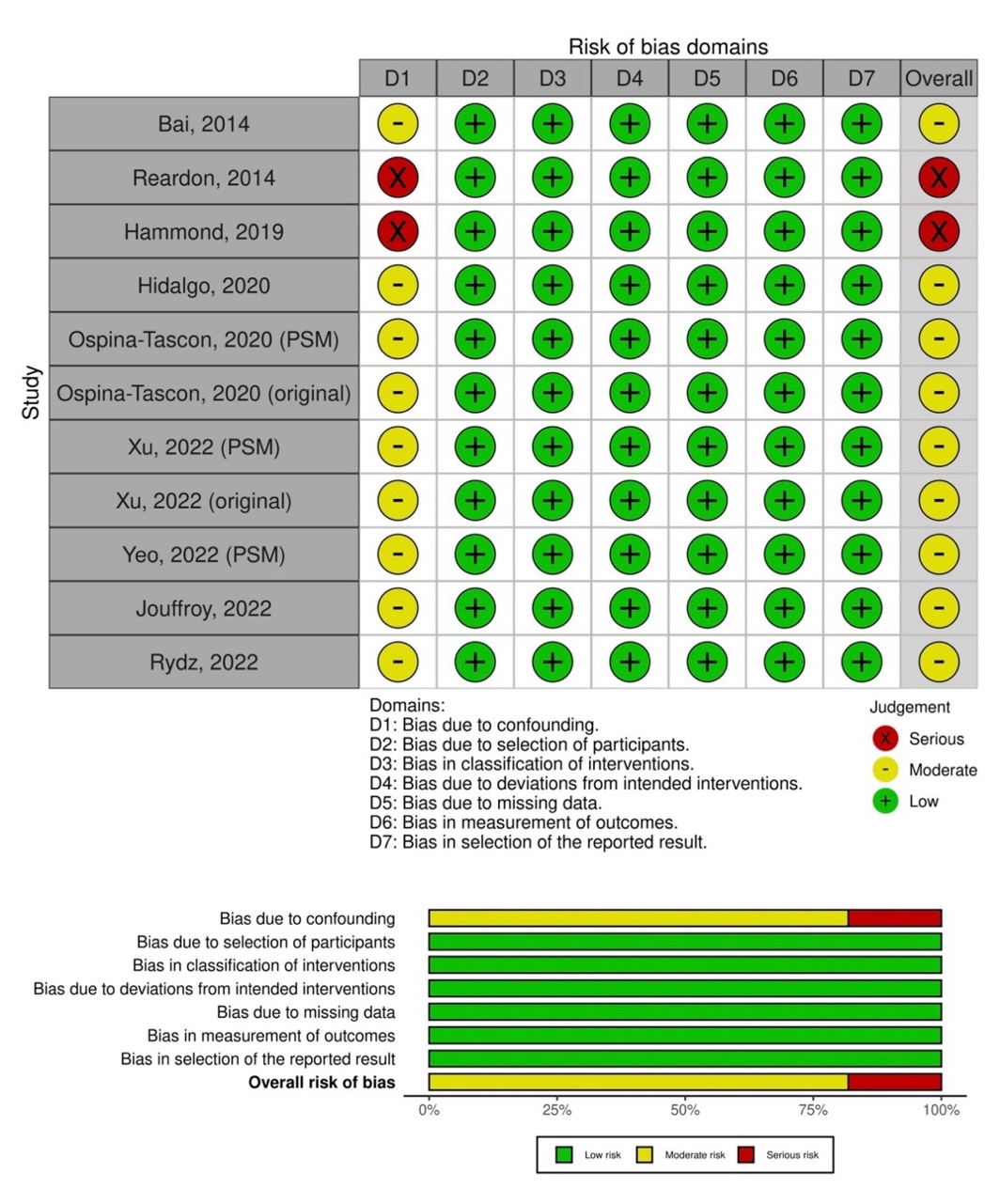

Supplement: Supplementary file 1 — Supplementary Material 1: Additional file 1: Table S1. Search strategy of PubMed. Table S2. Search strategy of Embase. Table S3. Search strategy of Cochrane. Figure S1. Forest plot pooling results from cohort studies (include quasi-experimental studies) and randomized controlled trials for short-term mortality. Figure S2. Forest plot pooling results from randomized controlled trials for short-term mortality. Figure S3. Forest plot pooling results from cohort studies (only include original) for short-term mortality. Figure S4. Forest plot pooling results from quasi-experimental studies for short-term mortality. Figure S5. Forest plot pooling results from cohort studies (include quasi-experimental studies) and randomized controlled trials for ICU length of stay. Figure S6. Forest plot pooling results from randomized controlled trials for ICU length of stay. Figure S7. Forest plot pooling results from cohort studies (only include original) for ICU length of stay. Figure S8. Forest plot pooling results from quasi-experimental studies for ICU length of stay. Figure S9. Meta-analysis result based on cohort studies (include quasi-experimental studies) and randomized controlled trials. Figure S10. Funnel plots of cohort studies (left) and randomized controlled trials (right). Figure S11. Forest plot pooling results from cohort studies (include quasi-experimental studies) and randomized controlled trials that exclude studies on vasopressin. Figure S12. Forest plot pooling results from cohort studies (include quasi-experimental studies) and randomized controlled trials that exclude studies with high risk of bias. Figure S13. Forest plot pooling results from randomized controlled trials and quasi-experimental studies that defined “time zero” as the onset of septic shock for short-term mortality. Figure S14. Forest plot pooling results from randomized controlled trials and quasi-experimental studies that defined “time zero” as the first fluid bolus for short-term mortality. Fig [file 13613_2025_1428_MOESM1_ESM.docx]
